# Dyke intrusion between neighbouring arc volcanoes responsible for 2017 pre-eruptive seismic swarm at Agung

**DOI:** 10.1038/s41467-019-08564-9

**Published:** 2019-02-14

**Authors:** Fabien Albino, Juliet Biggs, Devy Kamil Syahbana

**Affiliations:** 1 Centre for the Observation and Modelling of Earthquakes, Volcanoes and Tectonics (COMET), School of Earth Sciences, University of Bristol, Wills Memorial Building, Queens Road, Bristol, BS8 1RJ UK; 2 Center for Volcanology and Geological Hazard Mitigation (CVGHM), Jl. Diponegoro no. 57, Bandung, 40122 Indonesia

## Abstract

Forecasting explosive eruptions relies on using monitoring data to interpret the patterns and timescales of magma transport and mixing. In September 2017, a distal seismic swarm triggered the evacuation of around 140,000 people from Agung volcano, Bali. From satellite imagery and 3D numerical models, we show that seismicity was associated with a deep, sub-vertical magma intrusion between Agung and its neighbour Batur. This, combined with observations of the 1963 eruption which caused more than thousand fatalities, suggests a vertically and laterally interconnected system experiencing recurring magma mixing. The geometry of the 2017 dyke is consistent with transport from a deep mafic source to a shallow andesitic reservoir controlled by stresses induced by the topographic load, but not the regional tectonics. The ongoing interactions between Agung and Batur have important implications for interpretation of distal seismicity, the links between closely spaced arc volcanoes, and the potential for cascading hazards.

## Abstract (web summary)

Using seismic data and numerical modelling, here, the authors characterize the three-month period of unrest occurring prior to the 2017 Agung eruption (Bali, Indonesia). They observe a large uplift signal located at ~5 km from Agung summit corresponding to the emplacement of a 10 km deep magma intrusion between Agung edifice and Batur caldera, suggesting a potential magmatic connection between the two volcanic systems.

## Introduction

Signs that andesitic stratovolcanoes are reawakening after several decades of quiescence can include seismic swarms, release of volcanic gases and surface deformation and have the potential to inform eruption forecasts^1,2^. These signals can last from a few hours to many years but do not necessarily culminate in an eruption^3,4^, meaning a clear understanding of the timescales and patterns of magma transport through the upper crust is critical for eruption forecasts. Unfortunately, less than half of the world’s Holocene volcanoes are monitored^5^, with ground-based networks focussed on persistently or frequently active systems close to large populations. Conceptual models exist for basaltic volcanoes, where magmatic dykes solidify rapidly and subsequent eruptions require fresh intrusions^6,7^ and for persistently active andesitic systems, where the elevated magma supply maintains a long-lived conduit^8,9^. In comparison, relatively little is known about the magma pathways that form after decades of quiescence as these systems are generally considered a low priority for monitoring. In many cases, there is no pre-existing seismic network while in others, the network consists of too few stations or poorly maintained equipment.

Satellite geodetic data provide a complementary approach to understanding the drivers of volcanic unrest, which does not rely on ground-based logistics. InSAR images are produced every 6–12 days globally by the European Sentinel-1 constellation^10^, with even more frequent observations possible for specific targets by using multiple satellites^11^. There is a statistically significant link between satellite-detected surface deformation and eruption^12^ and modelling high-resolution images of surface deformation enables us to distinguish between earthquakes occurring on existing fault systems^13^, those associated with lateral magma propagation^14^ and those associated with inflation of a magmatic reservoir^15^ (but not necessarily whether through the input of fresh magma or volatiles^16^). The ability to resolve these processes in space and time is key to understanding the nature and connectivity of magmatic systems^17^ and the mechanisms driving volcanic unrest.

**Fig. 1:**
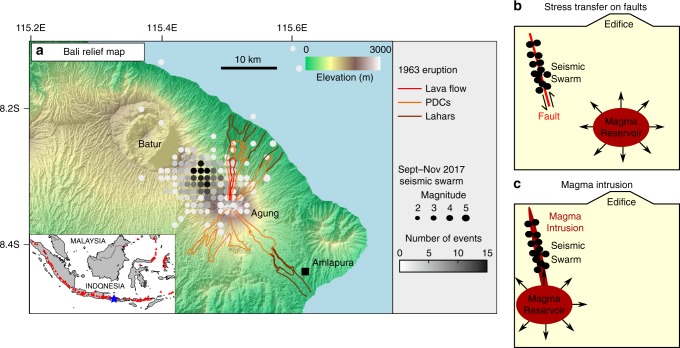
Location of the September−November 2017 seismic swarm recorded at Agung volcano prior to the eruption. **a** Topographic map of Bali island showing the volcanoes Agung and Batur, the density of seismic events (Meteorological, Climatological, and Geophysical Agency, BMKG) and the extent of the 1963 eruptive products^19^. Inset shows the location of the studied area (blue star) along the Indonesian volcanic arc. The spatial offset between the seismicity and the edifice can be explained by **b** stress transfer on existing faults from a central magmatic system or **c** stress changes associated with the propagation of a distal magma intrusion

The 2017 seismic swarm at Agung volcano, Bali is significant both socially and scientifically. The history of large and deadly eruptions^18,19^ followed by several decades of quiescence, combined with high population exposure and the economic importance of local tourist and agricultural industries resulted in intense political pressure on the response. The spatial and temporal pattern of seismicity, detected by the regional network of the Indonesian Meteorological, Climatological, and Geophysical Agency (BMKG) (http://inatews.bmkg.go.id/) and shown in Fig. 1a, suggests that the traditional model of elastic inflation of a single magma chamber prior to eruption is overly simplistic, and a more complex set of volcano−tectonic interactions occurred. Firstly, the seismicity was located midway between Agung and the neighbouring volcano, Batur caldera and was initially interpreted as triggered events on a nearby fault system (Fig. 1b), but could also indicate lateral magma transport (Fig. 1c). Secondly, there was a 10-week delay between the onset of seismicity and the first significant eruption.

Transcrustal magma systems facilitate the vertical transport of magma, but with the exception of major dyke intrusions in extensional tectonic settings, geophysical examples of lateral magma transport in the upper crust are limited to distances <10–15 km^20,21^. However, tephrochronology records simultaneous eruptions of volcanic systems spaced over tens of kilometres, particularly in extensional environments^22,23^ and a few historical examples suggest such connections also occur in arc settings^24,25^. Simultaneous eruptions of neighbouring volcanoes would present a heightened challenge for disaster management^26^, but the identification of precursory characteristics is hampered by the lack of well-monitored examples. In this study, we use satellite observations to investigate the mechanisms driving distal seismic swarms, combined with petrological constraints to provide new insights into the structure and connectivity of magmatic systems.

## Results

### Timeline

**Fig. 2:**
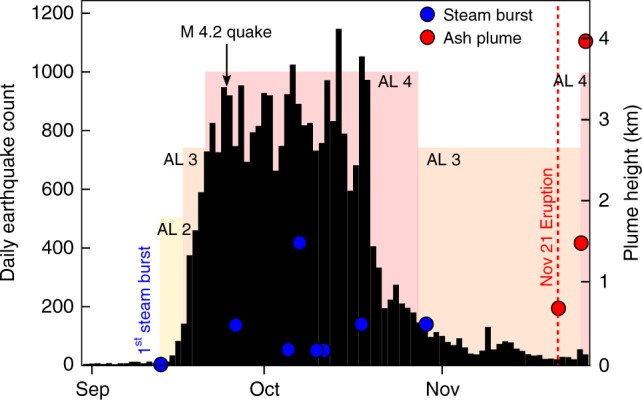
Timeline of observations during the 2017 seismic crisis at Agung volcano. Black histogram shows the daily number of seismic events provided by the Indonesian Center for Volcanology and Geological Hazard Mitigation (CVGHM). Blue and red circles indicate the height of steam bursts and ash plumes, respectively. The coloured background indicates the different Alert Levels provided by the CVGHM during the crisis

The volcanoes Agung and Batur are located 18 km apart on the Indonesian island of Bali (Fig. 1a). After several decades of quiescence, the local seismic network, operated by the Indonesian Center for Volcanology and Geological Hazard Mitigation (CVGHM), detected a rapid increase in seismicity between 14 and 22 September 2017 (Fig. 2). On 22 September, the CVGHM increased the Alert Level for Agung to its maximum value of 4 (CVGHM Press Release, https://magma.vsi.esdm.go.id), triggering the evacuation of around 140,000 people from within 9–12 km of the summit. Seismic activity remained high with local seismic stations recording >700 events per day for 4 weeks, before dropping significantly to <300 events per day in late-October (Fig. 2). This seismic crisis was accompanied by emissions of steam in the crater and intermittent plumes reaching few hundreds of metres high (Fig. 2). During late-October, the seismic event rate sharply dropped, which led the CVGHM to decrease the Alert Level to 3 on 29 October. Despite the apparent decrease in seismic activity, Agung started to erupt on 21 November 2017. The eruption initiated with a phreatomagmatic phase followed a few days later by a pure magmatic phase associated with the emission of successive ash columns that rose up to 4 km above the crater rim.

The greatest density of seismic events was located midway between Agung and Batur (Fig. 1a), consistent with seismic travel times from the four local short-period stations (two on the south flank of Agung and two within the Batur caldera). The distinction between distal seismic events triggered on tectonic faults by stress changes associated with a central magmatic system (Fig. 1b) and those caused by off-axis magma intrusion (Fig. 1c) are significant for eruption forecasting^2^ and understanding the structure of the magmatic system^17^. Unfortunately, a detailed analysis of the spatio-temporal trends in seismicity is not possible with the available network. In this study, we focus on the use of high-resolution satellite geodetic data, which provides an alternative source of information that is especially valuable where ground-based monitoring is limited.

### Pre-eruptive ground deformation

We use Sentinel-1 satellite radar data to measure surface deformation in Bali from 15 April to 21 November 2017, combining 12-day interferograms from ascending and descending tracks to constrain the geometry of the displacement field. Atmospheric effects, particularly those associated with variations in stratified tropospheric water vapour, are particularly strong in humid tropical regions such as Bali^27^. Atmospheric artefacts are clearly visible in the raw interferograms with the root mean square variability ranging from 0.9 to 3.2 cm. We test possible correction approaches (see Methods) and apply an Iterative Tropospheric Decomposition (ITD)^28,29^ to a high-resolution weather model to separate stratified and turbulent signals from tropospheric total delays, and generate line-of-sight atmospheric delay maps at the time of image acquisition.

**Fig. 3:**
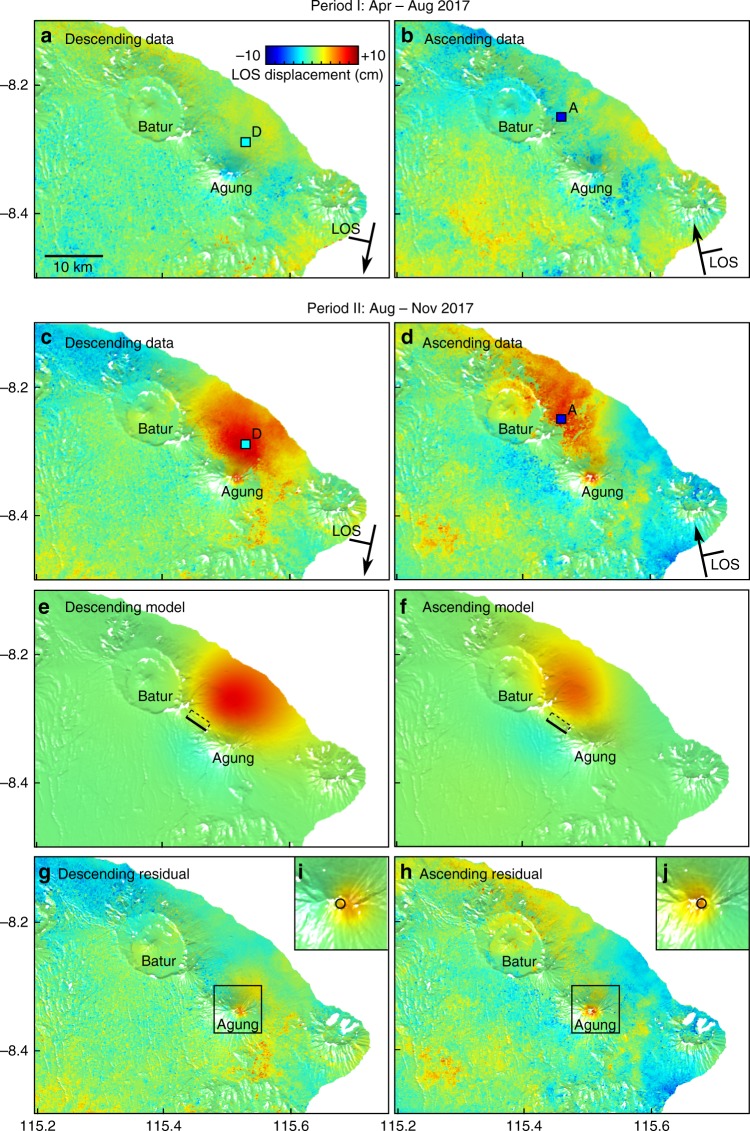
Ground deformation signals detected around Agung and Batur volcanoes. **a**–**d** Surface deformation maps are derived from stacked and corrected Sentinel-1 interferograms (see Methods). **a**, **b** period I from April to August 2017, showing no significant ground displacements and **c**, **d** period II from August to November 2017, showing a wide ground uplift on the northern flank of Agung. **e**, **f** Model displacement for the period II: best-fit model is a deep dyke intrusion located between Agung and Batur (black rectangle with bold line being the top edge). **g**, **h** Residual displacements obtained after removing the signal induced by the intrusion model. Both tracks show a residual uplift signal on Agung summit (black square), which has been modelled by **i**, **j** a small spherical source located 1 km below the edifice summit (~2 km above sea level)

**Fig. 4:**
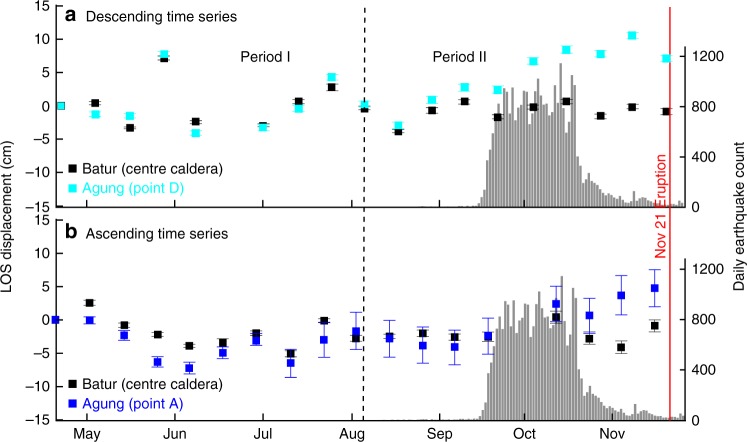
Time series of ground deformation at Agung and Batur volcanoes. The time series at Agung are plotted at the location of the maximum cumulative los displacements for **a** descending (light blue) and **b** ascending data set (dark blue), (see points D and A in Fig. 3c, d). The time series of the centre of Batur (black) is shown as a reference as it is not deforming during the time period. The central value and the uncertainty (1*σ*) at each epoch correspond to the mean and the standard deviation of line-of-sight displacements calculated for a 21 × 21 window

Initially, we subdivide our data set in two 4-month periods: period I pre-dates the seismic crisis (from mid-April to August) and shows no net deformation (Fig. 3a, b) while period II covers the period prior to the onset of eruption (from August to mid-November) and shows ground deformation on north flank of Agung (Fig. 3c, d). In the descending stack, the deforming region appears roughly circular with los displacement of ~8 cm located 6–7 km N-NE from the volcano summit (Fig. 3c). In the ascending stack, the deforming region appears elongated NW-SE extending from the north flank of Agung to the Eastern rim of Batur caldera, with a maximum line-of-sight (los) displacement of ~5 cm located 12 km N-NW from the summit (Fig. 3d). This apparent discrepancy is caused by the difference in viewing geometries between ascending and descending tracks and indicates a significant horizontal component to the displacement field. For both tracks, the line-of-sight displacement increased continuously with time between September and November 21st (Fig. 4). Maximum rates of ground displacements occurred at the peak of seismicity and reached about 60 and ~50 cm yr^−1^ for descending and ascending tracks, respectively.

### Source modelling

Next we investigate the geometry of the source responsible for causing the observed deformation. In cases of steep topography, elastic-half space models do not accurately reproduce deformation patterns caused by shallow sources^30^. The topography of Bali is steep and asymmetrical; there is a ridge joining Agung and Batur, the northern flank of which slopes steeply into the Bali Sea, while the southern flank is more gentle (Fig. 1a). We use the commercial Finite Element Modelling software COMSOL Multiphysics (https://www.comsol.com)^31^ to produce forward and inverse models of the surface deformation caused by pressurized sources beneath the topography of Bali (see Methods).

Initially, we test whether a range of simple source geometries such as a sphere^32^ or rectangular dislocation^33^ are capable of reproducing the observed pattern of deformation. Sources directly beneath the summit of Agung are not able to recreate the offset of the deformation. For the spherical case, the best-fit is obtained for source located 11 km north of Agung (Supplementary Figure 1c,d) and is associated with root-mean-square errors (RMSE) of 1.2 and 1.6 cm for descending and ascending tracks, respectively (Supplementary Figure 1e,f). The presence of a magma reservoir at this location is inconsistent with the distribution of the volcanic centres or location of the seismic swarm. In contrast, a dyke-shaped intrusion between Agung and Batur reproduces the general characteristics of the deformation patterns well (Fig. 3e, f) with RMSE of 0.96 and 1.5 cm for descending and ascending tracks, respectively (Fig. 3g, h). The best-fitting dyke model (see Methods) locates the intrusion midway between Agung and Batur at a depth of ~10 ± 0.3 km below sea level, with a strike of 129 ± 2° and a dip of 63 ± 2° (Supplementary Table 3). The dimensions of the dyke and the magma overpressure are poorly constrained, but all parameter combinations produce a total volume change of ~48 × 10^6^ m^3^. The total seismic moment release of the earthquakes detected by the regional network (4.3 × 10^16^ Nm) is an order of magnitude less than the geodetic moment release (5.7 × 10^17^ Nm) as is typical for dyke intrusions^14,34^. The ratio between the inferred intruded volume and the cumulative seismic moment is similar to that found for other volcano-tectonic earthquake swarms^2^.

After removing the deformation related to the dyke intrusion, a small concentric pattern of uplift remains at the summit of Agung in both ascending and descending tracks (Fig. 3g, h). The model that best fits these residual displacements is a spherical source located 1 km below the summit (~2 km above sea level) with a positive volume change of 0.37 × 10^6^ m^3^ (Fig. 3i, j). The very shallow depth suggests a hydrothermal rather than magmatic origin, with inflation induced by the pressurization of the hydrothermal system in response to the magma migration towards the surface.

### Temporal evolution

The temporal evolution of seismic and geodetic signals can provide valuable information regarding the mechanism and timescale of magma transport. Evidence of lateral dyke migration over timescales ranging from hours to few weeks has been found on well-monitored volcanoes in Hawai’i^35^, Iceland^14^ and Japan^36,37^. In case of a simple hydraulic connection between a magmatic reservoir and an intrusion, analytical models predict an exponentially decaying rate of the intruded volume with observed time constants varying from hours to days^35–38^. In contrast, some dyke intrusions show evidence of initial lateral propagation followed by opening over several weeks, perhaps due to the existence of a stress barrier^39^.

The temporal evolution of the 2017 dyke intrusion has significant implications for the conceptual model of the Agung−Batur system, specifically whether the dyke propagated vertically from a deep source or laterally from beneath Agung. Unfortunately, the limited seismic network that was operating during the early phases of the 2017 swarm at Agung cannot provide locations with sufficient accuracy to track the magma migration, and thus we focus on satellite geodetic data instead. We subdivide the descending InSAR data into a set of five cumulative stacks (Supplementary Figure 2a) and perform a non-linear inversion for each stack. We fix the orientation (strike, dip) and the centre depth of the intrusion based on the previous inversion result and solve for the position and the volume change of the dyke (Supplementary Table 4, Supplementary Figure 2b). For each time-step, the inferred model explains the data well, with RMSE ranging between 1.1 and 1.4 cm (Supplementary Figure 2c).

**Fig. 5:**
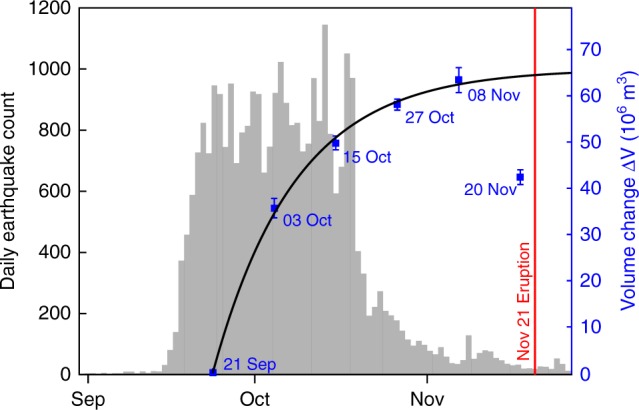
Relationship between the rate of seismic activity and the emplacement of the magma intrusion. Time series show the daily number of seismic events (grey) and the volume history of the intrusion (blue). The volume changes, Δ*V*_t_, have been inferred from the time-step inversion of InSAR descending stacks by using an inverse finite element model (see Methods). Values are well fit by an exponential decay: Δ*V*_t_ = Δ*V*_∞_(1 − *e*^−t/*τ*^), with an asymptotic volume change Δ*V*_∞_ = 66 ± 5 × 10^6^ m^3^ and timescale *τ* = 16 ± 4 days

The best-fitting source parameters show evidence for an exponentially decaying increase in dyke volume, but do not provide robust evidence of either lateral or vertical propagation. We conclude that significant propagation took less than the 12-day interval between SAR images, but the volume change continued for at least 48 days (until 8 November with Δ*V* = 63.4 × 10^6^ m^3^) (Fig. 5). The total dyke volume inferred on 20 November 2017, 1 day before the eruption, is significantly less (20 × 10^6^ m^3^) than in the previous time-step and the decrease cannot be attributed to uncertainties. Plausible physical processes to explain a volume loss include magma cooling by solidification and/or degassing^40^ or magma withdrawal^14^ into a shallow compressible reservoir.

The rate of volume change was greatest in the first 12 days and gradually decreased through time, providing a good fit to a model of exponential decay^35,38^ for an asymptotic volume change of Δ*V*_∞_ = 66 × 10^6^ m^3^ and a timescale of *τ* = 16 days (Fig. 5). This timescale is much greater than the several hours observed for intrusions at Kilauea or Miyakejima^38^, but the magma migration was still too rapid to be detected by Sentinel-1 InSAR timeseries. Seventy-five per cent of the total volume of the intrusion was emplaced between 21 September and 15 October, which corresponds to the period of heightened seismicity. The correlation between the volume history of the intrusion and seismic rate confirms once again the link between the seismic swarm and the dyke intrusion.

### Stress field

Dyke swarms between basaltic volcanoes are common in rift settings where the extensional stresses favour vertical dyke propagation^41^ but in subduction settings, horizontal compressive stresses favour the formation of sills^42,43^. The 2017 dyke opening direction (*σ*_3_) of 039 ± 1.5° is not consistent with either the convergence between the Australian and Sunda plates at an azimuth of ~167°^44^, or the direction of maximum horizontal stress (SH) inferred from nearby earthquakes of 021°–039°^45^.

**Fig. 6:**
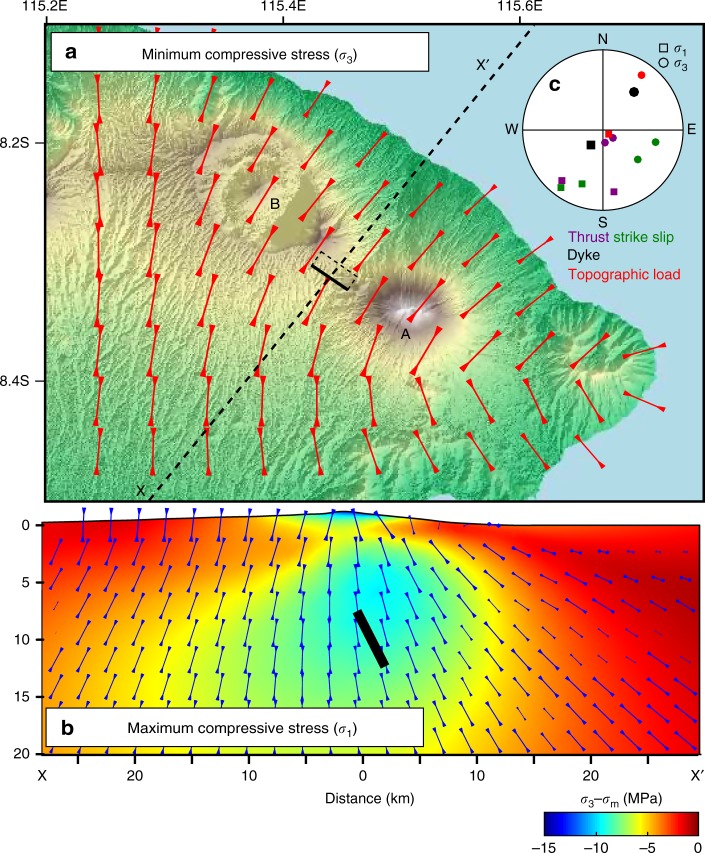
Effect of the topographic load on the emplacement of the magma intrusion. **a** Orientation of the minimum compressive stress (*σ*_3_) at 10 km bsl (equivalent to the depth of the dyke). The small black rectangle represents the dyke (bold line being the top edge). **b** Depth profile across the dyke (see location in (**a**)) showing the orientation of the maximum compressive stress *σ*_1_ as well as the minimum deviatoric stress (*σ*_3_ − *σ*_m_), with *σ*_m_ being the mean stress. **c** Stereographic projection showing the direction of *σ*_1_ and *σ*_3_ for the tectonic stresses (focal mechanism of thrust and strike-slip earthquakes), the inferred dyke (FEM-based inversion of InSAR data) and the topographic loading (FEM stress model)

However, local stresses associated with pressurized magma reservoirs and edifice topography can produce radial and/or circumferential intrusions^46–48^. These radial intrusions are often clustered in a preferential direction through interactions with tectonic stress fields, pre-existing structures or other topographic loads^49,50^. The stress field associated with the loading of the edifices of Agung and Batur (see Methods) has the following characteristics: (1) the minimum compressive stress, *σ*_3_, is oriented at 035° at 10 km depth (Fig. 6a), (2) the maximum compressive stress, *σ*_1_, is sub-vertical, favouring the intrusion of dykes over sills, and (3) the minimum deviatoric stress is highly negative (tension) midway between the two edifices (Fig. 6b). These features of the stress field are consistent with the orientation and the location of the inferred dyke (Fig. 6c).

## Discussion

The 2017 dyke intrusion occurred midway between the edifices of Agung and Batur at depths of 7–13 km suggesting a physical connection between the two magmatic systems, and between deep and shallow reservoirs. The hypothesis of a vertically and laterally extensive magma system is supported by the historical observations of simultaneous eruptions at Batur and Agung and similarities in storage depths and eruptive compositions. Agung has experienced one eruption greater than VEI2-3 per century over the past 5000 years^19^, including the VEI 5 eruption in 1963, which was one of the largest of the twentieth century and produced pyroclastic flows and lahars, killing more than 1000 people^51^. Batur has produced at least nine basaltic flows between 1849 and 2000. The most voluminous of these occurred in September 1963, coincident with the eruption of Agung, and began a decade-long period of small eruptions^52,53^.

**Fig. 7:**
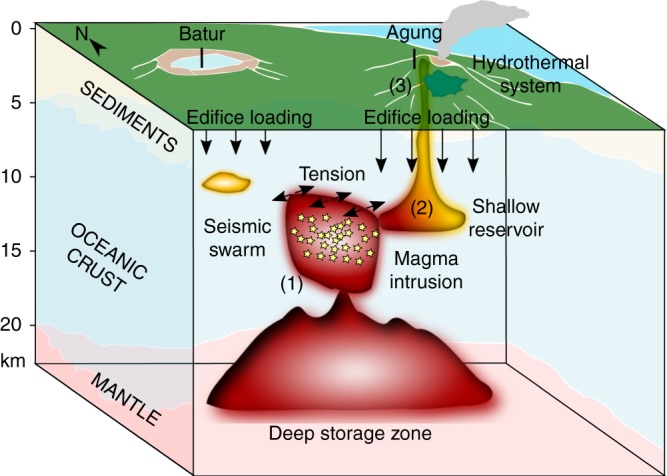
Conceptual model of the magmatic systems below Agung and Batur volcanoes. The model is based on geophysical observations from 2017 and petrological studies of previous eruptions^18,19,54^. The numbers 1–3 indicate the different processes that occurred prior to the 2017 eruption: (1) the emplacement of a magma intrusion between Agung and Batur, which induced seismicity and ground deformation; (2) mixing between mafic magma from the intrusion and a more differentiated magma in a pre-existing shallow reservoir; (3) ascent of the mixed magma causing pressurization of the hydrothermal system and initiation of the eruption

Thermobarometry of the 1963 eruptive products indicates at least two different levels of magma storage^54^: (i) a deep reservoir at 10–30 km depth, likely corresponding to the Moho, and (ii) a shallow reservoir at 3–7 km depth, which corresponds to the lithological transition between the sediments and the oceanic crust. These depths are consistent with the top and bottom of the 2017 dyke, consistent with magma transport between deep and shallow reservoirs (Fig. 7). Moreover, the temporal evolution of the intruded volume supports a hydraulic connection between the dyke and the deep reservoir.

The orientation of the 2017 intrusion was controlled by the loading stresses of the volcanic edifices rather than tectonic forces, which underlines the importance of local stress during magma transfer. Topographically controlled lateral magma transport could explain the simultaneous eruptions of Agung and Batur in 1963, but also records of simultaneous eruptions at other pairs of arc volcanoes, such as Katmai and Novarupta, Karymsky and Academy Nauk (Kamchatka)^55^ or Fuego and Agua (Guatemala)^26^.

Magma mixing is often proposed as a trigger mechanism of explosive eruptions^56–59^ including Agung’s 1963 eruption^18,19^. Observations of texturally complex mineral assemblages from the 5000-year tephrastratigraphic record at Agung suggest that all the explosive eruptions result from intrusions of basaltic magmas into basaltic andesitic to andesitic reservoirs^19^. Extending this conceptual model of recurring magmatic processes could explain several features of the 2017 sequence. In this scenario, the observed dyke was fed by a deep mafic source (see (1) in Fig. 7) and intersected a pre-existing shallow reservoir containing partially crystalline andesite or basaltic andesite magma (see (2) in Fig. 7). Timescales of magma mixing typically range from a few days^57^ to a few months^59^, consistent with the month delay between the arrest of the dyke in mid-October and the start of the eruption on 21 November. We do not detect any deformation associated with the 5–7 km shallow reservoir but magma mixing may have produced negligible pressure changes if the magma is (1) gas-rich and highly compressible^60^ consistent with observations of high co-eruptive SO_2_ fluxes in 1963 and 2017^18^, and/or (2) an open system consistent with sulphur-rich emissions and our observation that the shallow hydrothermal system was pressurizing from early November (see (3) in Fig. 7). However, petrological analysis of the 2017 erupted products will be required to confirm this hypothesis.

The 2017 Agung unrest sequence has scientific implications for the understanding of the processes and timescales of magma transport in the upper crust, particularly regarding the connections between reservoirs of different compositions and between different volcanic systems. The stress conditions required for dyke intrusion were generated by the topographic load of closely spaced edifices rather than the tectonic environment and are not unique to this example. Furthermore, we identify practical considerations for the interpretation of distal seismicity during volcanic unrest, and highlight the utility of satellite geodetic observations in distinguishing between conceptual models. The prospect of cascading hazards caused by simultaneous eruptions of neighbouring volcanic systems has largely been neglected, but deserves further attention. These findings have important consequences for evaluating future eruptive scenarios and for improving eruption forecasting globally.

## Methods

### InSAR processing and atmospheric corrections

**Fig. 8:**
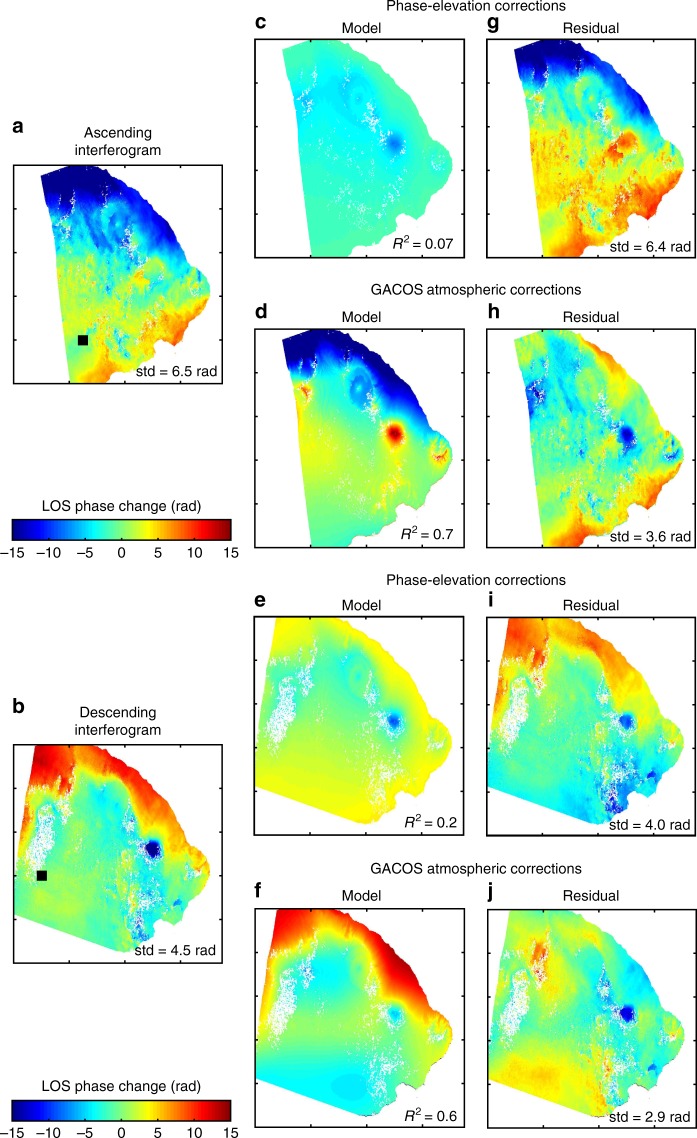
Effect of atmospheric corrections. We compare between two different methods to correct atmospheric delays. **a**, **b** uncorrected 12-day interferograms for **a** ascending (20170813–20170825) and **b** descending (20170828–20170909) tracks. The black square locates the reference window where deformation is considered to be zero; **c**–**f** tropospheric delays estimates using **c**, **e** a linear relationship between the elevation and the phase or **d**, **f** the weather-model corrections; **g**–**j** corresponding corrected interferograms

We process Sentinel-1 Interferometric Wideswath (IW) images between April and November 2017 with the LiCSAR/GAMMA software^61^ and produce 12-day interferograms: 17 from ascending track (156A) and 17 from descending track (32D) (Supplementary Tables 1–2). Topographical corrections are applied using the SRTM 1 Digital Elevation Model (DEM) and pixels with coherence <0.25 were masked. We reference the interferograms to a 21 × 21 pixel low relief area located far from the volcanoes (Fig. 8a, b).

Bali lies in the tropics, meaning there is significant variability in tropospheric water vapour. This causes phase delays in interferograms, particularly in regions of high relief such as volcanic edifices^27,62^. We assess two methods of atmospheric correction: phase-elevation correlation^63,64^ and global weather models^28,29,62^, and illustrate the approaches using example interferograms from ascending and descending tracks (Fig. 8). The phase correlation method provides a poor fit to the data, particularly along the coastal areas, with a coefficient of determination (*R*^2^) of 0.07 and 0.2 for the ascending and the descending interferograms respectively (Fig. 8c, e). The standard deviation of the residuals remains high and atmospheric artefacts present on the NE coastline are not corrected (Fig. 8g, i).

We then apply a weather-model approach using the Generic Atmospheric Correction Online Service (GACOS)^28,29^. The corrections are based on a global weather model at high resolution (0.125-degree and 6-h resolutions) provided by the European Centre for Medium-Range Weather Forecasts (ECMWF) and interpolated to the time of each acquisition (Supplementary Tables 1−2) using an ITD^28,29^. The phase delays are then converted from zenith to the appropriate line-of-sight (los). The correlation coefficient between raw interferograms and GACOS corrections is much higher than for the phase-elevation approach (*R*^2^ = 0.7 and *R*^2^ = 0.6 for ascending and descending tracks respectively) (Fig. 8d, f). The large artefacts on the NE coast are well represented and the correction reduced the standard deviation of the interferograms by 35–45% (Fig. 8h, j).

We conclude that atmospheric artefacts are significant in this region and that the weather-model corrections are more effective than the simpler phase-elevation correlation method. We apply weather-model corrections to the entire data set and stack the corrected interferograms to further reduce the influence of turbulent atmospheric effects^65^. Furthermore, this suggests that previous InSAR observations of deformation at Agung^66^ should be re-analysed using a similar approach.

### Modelling

We model the pre-eruptive displacement and stress fields with the commercial Finite Element Modelling (FEM) software COMSOL Multiphysics. We use a 100 × 100 × 50 km domain designed to limit boundary effects (fixed bottom and lateral boundaries; free top boundary) and the 90 m SRTM DEM to represent surface topography. We use an adaptive mesh at the free surface of the model, with the mesh size ranges from 5 km far from the volcanoes (no deformation) to 100 m between Agung and Batur (high deformation gradient). We assume the host rock is an isotropic, homogeneous elastic medium with a Young’s modulus of 30 GPa and Poisson’s ratio of 0.25, which are consistent with values inferred from seismic velocity in the Sunda arc^67^ and rock experiments on igneous rocks^68^.

We model the deformation pattern by representing the magmatic sources as cavities and calculating the stress perturbations relative to lithostatic by applying a constant overpressure to their boundaries. The dyke-like source model has nine parameters: the position of the dyke relative to the summit of Agung (*X*, *Y*) and the depth (*Z*) relative to sea level; the length (*L*) and height (*H*) of the dyke; the orientation of the dyke defined by strike (*α*) and dip (*δ*), and the dyke overpressure (Δ*P*). To allow for sensible meshing, we set the initial thickness to 100 m and verify that this choice has no significant influence on the result. We initially test several candidate geometries using forward models, then jointly invert the ascending and descending final stacks (Fig. 3c, d) to find the best-fitting geometry for a dyke-like intrusion, using an objective function that takes into account the misfit to both tracks of data. The InSAR data is sub-sampled according to the mesh and the misfit is calculated from the numerical integration of each FEM element at the surface.

Markov Chain Monte-Carlo (MCMC) inversions currently are too computationally expensive to be used with Finite Element Models as about 100,000 simulations are typically required to characterize the a-posteriori probability density function for each model parameter^69^. For an analytical model (1 simulation per second), this requires about a day, but for a 3D FEM (1 simulation per minute), this would require about 70 days of computation. We therefore use hybrid optimization scheme combining (1) a random search (Monte-Carlo) for the initialization of the parameter values and (2) a downhill simplex method (Nelder−Mead) for convergence towards the optimal parameters. The downhill simplex (Nelder−Mead) method is applicable to non-linear optimization problems for which the derivatives are unknown. The method uses the concept of a simplex, which is a polytope of *n* + 1 vertices in *n* dimensions (e.g. a triangle on a plane or a tetrahedron in 3D) to find the minimum of the objective function. The optimization consists of a series of steps where the point of the simplex with the largest objective function moves towards a lower point. The limitation of the Nelder−Mead technique is that it may converge to a local minimum and the result can be strongly dependent on the set of parameters chosen for the first simplex. To avoid this, we run a series of Nelder−Mead optimizations with initializations defined by a Monte-Carlo exploration of the parameter space.

**Fig. 9:**
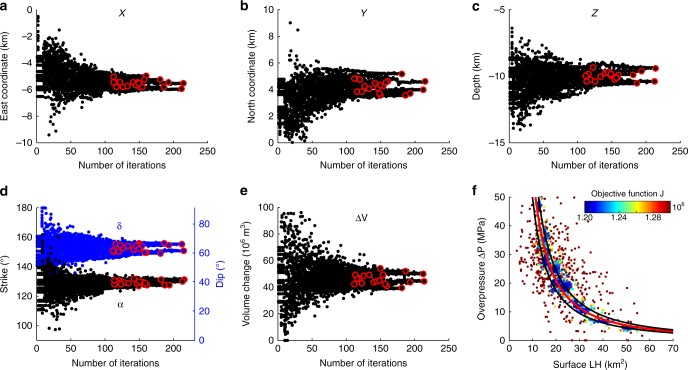
Parameters convergence and trade-off for the intrusion model. The non-linear joint inversion of ascending and descending InSAR cumulative stacks is performed using COMSOL Multiphysics. The convergence is obtained for **a** East coordinate, **b** North coordinate, **c** Centre depth, **d** Strike and Dip and **e** Volume change. Red circles correspond to the optimal values found after the set of 20 Nelder−Mead optimizations: the average and standard deviation values for each parameter are shown in Supplementary Table 3. **f** Parameter trade-off between the surface area of the intrusion and the magma overpressure. The red line shows a power-law function corresponding to the optimal volume change of 47.6 × 10^6^ m^3^ and the black lines indicate the 2*σ* uncertainty

We initially perform 1000 random-search simulations and the models with the smallest objective function are selected and used for the initial simplex. After each Nelder−Mead optimization, we calculate the mean and the standard deviation between the final values obtained for each parameter (red circles in Fig. 9). A new Nelder−Mead optimization is performed as long as the standard deviation remains above 10% of the mean value, and only 20 Nelder−Mead simulations are required for most of the model parameters to meet the criterion (Supplementary Table 3). Our hybrid approach significantly reduces the computing time compared to an MCMC approach, as in total only 4000 forward models were required for the optimization. For the inversion of the temporal subdivisions, we reduce the time even further by using the best-fitting dyke intrusion model for the cumulative displacement to initialize the inversion and a downhill simplex approach to search for nearby minima.

The convergence of the source parameters is shown in Fig. 9. The ensemble model reproduces the deformation pattern with an rms misfit of 0.96 and 1.5 cm for both descending and ascending stacks, respectively. From the inversion, three parameters (length *L*, height *H* and magma overpressure Δ*P*) did not converge due to a trade-off between the surface of the dyke and the magma overpressure (Fig. 9f). A small dyke with a large overpressure will cause the same surface displacements as a large dyke with low overpressure. This relationship can be described by a power-law function and the fit to the data shows that the optimal models are associated with a constant magma volume change (Δ*V*) rather than a constant intrusion size.

For the shallow reservoir model, we invert the residual displacement assuming a spherical source directly beneath the summit of Agung, described by two parameters: centre depth and volume change (Fig. 3i, j). This reduces the rms misfit of the residual displacements by 1–2 mm for ascending and descending tracks.

To estimate the local stress field associated with topographic loading, we apply a body load corresponding to the topography above sea level^70^ using the same model configuration as described for the deformation models (Fig. 6).

## Supplementary information

Supplementary Information

## Data Availability

Seismic event counts are acquired from the Agung seismic network operated by the Center for Volcanology and Geological Hazard Mitigation (CVGHM) and are available from the co-author (D.K.S.). InSAR processed data that support the findings of this study are freely available on the COMET-LiCS Sentinel-1 InSAR portal (http://comet.nerc.ac.uk/COMET-LiCS-portal). Atmospheric corrections maps can be requested via the Generic Atmospheric Correction Online Service for InSAR (GACOS) (http://ceg-research.ncl.ac.uk/v2/gacos). Numerical models and non-linear inversion have been performed using the software COMSOL Multiphysics (https://www.comsol.com) and source files are available from the corresponding author (F.A.) upon reasonable request.

## References

[1] Sparks R, Biggs J, Neuberg J (2012). Monitoring volcanoes. Science.

[2] White R, McCausland W (2016). Volcano-tectonic earthquakes: a new tool for estimating intrusive volumes and forecasting eruptions. J. Volcanol. Geotherm. Res..

[3] Phillipson G, Sobradelo R, Gottsmann J (2013). Global volcanic unrest in the 21st century: an analysis of the first decade. J. Volcanol. Geotherm. Res..

[4] Passarelli L, Brodsky EE (2012). The correlation between run-up and repose times of volcanic eruptions. Geophys. J. Int..

[5] Brown, S. K. et al. in *Global volcanic hazard and risk*, 81–172 (Cambridge University Press, Cambridge, 2015).

[6] Sigmundsson, F. *Iceland Geodynamics: Crustal Deformation and Divergent Plate Tectonics* (Springer Science & Business Media, Chichester, 2006).

[7] Poland MP (2015). Magma supply, storage, and transport at shield-stage. Charact. Hawaii. Volcanoes.

[8] Voight B (1999). Magma flow instability and cyclic activity at Soufriere Hills volcano, Montserrat, British West Indies. Science.

[9] Denlinger RP, Hoblitt RP (1999). Cyclic eruptive behavior of silicic volcanoes. Geology.

[10] Anantrasirichai, N., Biggs, J., Albino, F., Hill, P. & Bull, D. Application of machine learning to classification of volcanic deformation in routinely-generated InSAR data. *J. Geophys. Res.: Solid Earth*, 10.1029/2018JB015911 (2018).

[11] Pritchard M (2018). Towards coordinated regional multi-satellite InSAR volcano observations: results from the Latin America pilot project. J. Appl. Volcanol..

[12] Biggs J (2014). Global link between deformation and volcanic eruption quantified by satellite imagery. Nat. Commun..

[13] Ebmeier SK (2016). Shallow earthquake inhibits unrest near Chiles-Cerro Negro volcanoes, Ecuador-Colombian border. Earth Planet. Sci. Lett..

[14] Sigmundsson F (2015). Segmented lateral dyke growth in a rifting event at Bárdarbunga volcanic system, Iceland. Nature.

[15] Parks MM (2012). Evolution of Santorini Volcano dominated by episodic and rapid fluxes of melt from depth. Nat. Geosci..

[16] Hill DP (2017). Long Valley Caldera-Mammoth Mountain unrest: the knowns and the unknowns. Elements.

[17] Cashman KV, Sparks RSJ, Blundy JD (2017). Vertically extensive and unstable magmatic systems: a unified view of igneous processes. Science.

[18] Self S, King AJ (1996). Petrology and sulfur and chlorine emissions of the 1963 eruption of Gunung Agung, Bali, Indonesia. Bull. Volcanol..

[19] Fontijn K, Costa F, Sutawidjaja I, Newhall CG, Herrin JS (2015). A 5000-year record of multiple highly explosive mafic eruptions from Gunung Agung (Bali, Indonesia): implications for eruption frequency and volcanic hazards. Bull. Volcanol..

[20] Biggs J, Robertson E, Cashman K (2016). The lateral extent of volcanic interactions during unrest and eruption. Nat. Geosci..

[21] Ebmeier S (2018). Synthesis of global satellite observations of magmatic and volcanic deformation: implications for volcano monitoring & the lateral extent of magmatic domains. J. Appl. Volcanol..

[22] Cooper GF, Wilson CJ, Millet MA, Baker JA, Smith EG (2012). Systematic tapping of independent magma chambers during the 1 Ma Kidnappers supereruption. Earth Planet. Sci. Lett..

[23] Scott SC, Skilling IP (1999). The role of tephrachronology in recognizing synchronous caldera-forming events at the Quaternary volcanoes Longonot and Suswa, south Kenya Rift. Geol. Soc., Lond., Spec. Publ..

[24] Hildreth, W. & Fierstein, J. *The Novarupta-Katmai Eruption of 1912: Largest Eruption of the Twentieth Century: Centennial Perspectives*. U.S. Geological Survey Professional Paper 1791 (U.S. Geological Survey, Reston, VA, 2012).

[25] Jay J (2014). Locating magma reservoirs using InSAR and petrology before and during the 2011–2012 Cordón Caulle silicic eruption. Earth Planet. Sci. Lett..

[26] Hutchison A, Cashman K, Williams C, Rust A (2016). The 1717 eruption of Volcán de Fuego, Guatemala: cascading hazards and societal response. Quat. Int..

[27] Ebmeier SK, Biggs J, Mather TA, Amelung F (2013). Applicability of InSAR to tropical volcanoes: insights from Central America. Geol. Soc. Lond. Spec. Publ..

[28] Yu C, Penna NT, Li Z (2017). Generation of real-time mode high-resolution water vapor fields from GPS observations. J. Geophys. Res.: Atmospheres.

[29] Yu C, Li Z, Penna NT, Crippa P (2018). Generic atmospheric correction model for Interferometric Synthetic Aperture Radar observations. J. Geophys. Res.: Solid Earth.

[30] Cayol V, Cornet FH (1998). Effects of topography on the interpretation of the deformation field of prominent volcanoes application to Etna. Geophys. Res. Lett..

[31] Hickey J, Gottsmann J (2014). Benchmarking and developing numerical finite element models of volcanic deformation. J. Volcanol. Geotherm. Res..

[32] Mogi K (1958). Relations between the eruptions of various volcanoes and the deformations of the ground surfaces around them. Bull. Earthq. Res. Inst..

[33] Okada Y (1985). Surface deformation due to shear and tensile faults in a half-space. Bull. Seismol. Soc. Am..

[34] Wright TJ (2006). Magma-maintained rift segmentation at continental rupture in the 2005 Afar dyking episode. Nature.

[35] Segall P, Cervelli P, Owen S, Lisowski M, Miklius A (2001). Constraints on dike propagation from continuous GPS measurements. J. Geophys. Res.: Solid Earth.

[36] Irwan M, Kimata F, Fujii N (2006). Time dependent modeling of magma intrusion during the early stage of the 2000 Miyakejima activity. J. Volcanol. Geotherm. Res..

[37] Morita Y, Nakao S, Hayashi Y (2006). A quantitative approach to the dike intrusion process inferred from a joint analysis of geodetic and seismological data for the 1998 earthquake swarm off the east coast of Izu Peninsula, central Japan. J. Geophys. Res.: Solid Earth.

[38] Rivalta E (2010). Evidence that coupling to magma chambers controls the volume history and velocity of laterally propagating intrusions. J. Geophys. Res.: Solid Earth.

[39] Biggs J, Amelung F, Gourmelen N, Dixon TH, Kim SW (2009). InSAR observations of 2007 Tanzania rifting episode reveal mixed fault and dyke extension in an immature continental rift. Geophys. J. Int..

[40] Caricchi L, Biggs J, Annen C, Ebmeier S (2014). The influence of cooling, crystallisation and re-melting on the interpretation of geodetic signals in volcanic systems. Earth Planet. Sci. Lett..

[41] Gudmundsson A, Andrew RE (2007). Mechanical interaction between active volcanoes in Iceland. Geophys. Res. Lett..

[42] Menand T (2011). Physical controls and depth of emplacement of igneous bodies: a review. Tectonophysics.

[43] Maccaferri F, Bonafede M, Rivalta E (2011). A quantitative study of the mechanisms governing dike propagation, dike arrest and sill formation. J. Volcanol. Geotherm. Res..

[44] Kreemer C, Blewitt G, Klein EC (2014). A geodetic plate motion and Global Strain Rate Model. Geochem., Geophys., Geosystems.

[45] Heidbach O, Rajabi M, Reiter K, Ziegler M (2016). World stress map 2016. Science.

[46] Nakamura K (1977). Volcanoes as possible indicators of tectonic stress orientation—principle and proposal. J. Volcanol. Geotherm. Res..

[47] Chadwick WW, Dieterich JH (1995). Mechanical modeling of circumferential and radial dike intrusion on Galapagos volcanoes. J. Volcanol. Geotherm. Res..

[48] Acocella V, Neri M (2009). Dike propagation in volcanic edifices: overview and possible developments. Tectonophysics.

[49] Fiske RS, Jackson ED (1972). Orientation and growth of Hawaiian volcanic rifts: the effect of regional structure and gravitational stresses. Proc. R. Soc. Lond. A.

[50] Walter TR, Klügel A, Münn S (2006). Gravitational spreading and formation of new rift zones on overlapping volcanoes. Terra Nova.

[51] Self S, Rampino MR (2012). The 1963–1964 eruption of Agung volcano (Bali, Indonesia). Bull. Volcanol..

[52] Surjo I (1981). Report on the volcanic activity in Indonesia during the period 1961–1963. Bull. Volcano Surv. Indonesia.

[53] Wheller G, Varne R (1986). Genesis of dacitic magmatism at Batur volcano, Bali, Indonesia: implications for the origins of stratovolcano calderas. J. Volcanol. Geotherm. Res..

[54] Geiger H (2018). Multi-level magma plumbing at Agung and Batur volcanoes increases risk of hazardous eruptions. Sci. Rep..

[55] Eichelberger JC, Izbekov PE (2000). Eruption of andesite triggered by dyke injection: Contrasting cases at Karymsky Volcano, Kamchatka and Mt Katmai, Alaska. Philos. Trans. R. Soc. Lond. Math. Phys. Eng. Sci..

[56] Sparks SR, Sigurdsson H, Wilson L (1977). Magma mixing: a mechanism for triggering acid explosive eruptions. Nature.

[57] Sigmarsson O (2011). Dynamic magma mixing revealed by the 2010 Eyjafjallajökull eruption. Solid Earth Discuss..

[58] Pallister JS, Hoblitt RP, Reyes AG (1992). A basalt trigger for the 1991 eruptions of Pinatubo Volcano?. Nature.

[59] Nakamura M (1995). Continuous mixing of crystal mush and replenished magma in the ongoing Unzen eruption. Geology.

[60] Kilbride BM, Edmonds M, Biggs J (2016). Observing eruptions of gas-rich compressible magmas from space. Nat. Commun..

[61] Wegmüller U (2016). Sentinel-1 support in the GAMMA software. Procedia Comput. Sci..

[62] Parker AL (2015). Systematic assessment of atmospheric uncertainties for InSAR data at volcanic arcs using large-scale atmospheric models: Application to the Cascade volcanoes, United States. Remote Sens. Environ..

[63] Remy D, Bonvalot S, Briole P, Murakami M (2003). Accurate measurements of tropospheric effects in volcanic areas from SAR interferometry data: Application to Sakurajima volcano (Japan). Earth Planet. Sci. Lett..

[64] Bekaert DPS, Hooper A, Wright TJ (2015). A spatially variable power law tropospheric correction technique for InSAR data. J. Geophys. Res. Solid Earth.

[65] Peltzer G, Crampé F, Hensley S, Rosen P (2001). Transient strain accumulation and fault interaction in the Eastern California shear zone. Geology.

[66] Chaussard E, Amelung F (2012). Precursory inflation of shallow magma reservoirs at west Sunda volcanoes detected by InSAR. Geophys. Res. Lett..

[67] Wagner D (2007). Joint inversion of active and passive seismic data in Central Java. Geophys. J. Int..

[68] Christensen NI (1996). Poisson’s ratio and crustal seismology. J. Geophys. Res.: Solid Earth.

[69] Bagnardi M, Hooper A (2018). Inversion of surface deformation data for rapid estimates of source parameters and uncertainties: a Bayesian approach. Geochem. Geophys. Geosyst..

[70] Albino F, Amelung F, Gregg P (2018). The role of pore fluid pressure on the failure of magma reservoirs: insights from Indonesian and Aleutian arc volcanoes. J. Geophys. Res.: Solid Earth.

