# Supplementary material for: Dyke intrusion between neighbouring arc volcanoes responsible for 2017 pre-eruptive seismic swarm at Agung
Source: Nat Commun. 2019 Feb 14;10:748. doi: 10.1038/s41467-019-08564-9 (PMC6376036; doi:10.1038/s41467-019-08564-9)
Supplement: Supplementary file 1 — Supplementary Information [file 41467_2019_8564_MOESM1_ESM.pdf]

Supporting Information for "Dyke intrusion between neighbouring arc volcanoes responsible for 2017 pre-eruptive seismic swarm at Agung, Albino et al."

Contents

1. Supplementary Tables 1 to 4
2. Supplementary Figures 1 to 2

Corresponding author: Fabien Albino, fa17101@bristol.ac.uk

7 **Supplementary Table 1.** Parameters of the Sentinel-1 interferograms for the ascending track.

Master Date	Slave Date	Baseline Length	Mean coherence
20170415	20170427	84.23	0.82
20170427	20170509	142.07	0.82
20170509	20170521	66.60	0.88
20170521	20170602	69.78	0.85
20170602	20170614	9.21	0.87
20170614	20170626	43.74	0.85
20170626	20170708	49.01	0.76
20170708	20170720	28.81	0.78
20170720	20170801	95.46	0.78
20170801	20170813	14.58	0.78
20170813	20170825	33.12	0.83
20170825	20170906	51.91	0.87
20170906	20170918	89.80	0.83
20170918	20171012	15.23	0.79
20171012	20171024	19.83	0.84
20171024	20171105	35.80	0.86
20171105	20171117	27.79	0.84

8 **Supplementary Table 2.** Parameters of the Sentinel-1 interferograms for the descending track.

Master Date	Slave Date	Baseline length	Mean coherence
20170418	20170430	53.73	0.74
20170430	20170512	47.39	0.74
20170512	20170524	125.09	0.69
20170524	20170605	106.58	0.69
20170605	20170629	51.30	0.73
20170629	20170711	32.76	0.78
20170711	20170723	19.84	0.75
20170723	20170804	75.34	0.74
20170804	20170816	27.49	0.82
20170816	20170828	26.37	0.83
20170828	20170909	35.53	0.79
20170909	20170921	18.55	0.81
20170921	20171003	23.90	0.81
20171003	20171015	39.08	0.75
20171015	20171027	79.97	0.76
20171027	20171108	108.02	0.81
20171108	20171120	47.25	0.74

Supplementary Table 3. Optimal parameters of the dyke intrusion given by the combined inversion of ascending and descending final stacks (Figure 3c,d). Mean and standard deviation values are derived from the 20 Nelder-Mead optimization. The X and Y locations are calculated from the summit of Agung, relative to 115.5 E, 8.34 S.

Parameter	Mean	Standard Deviation
East coordinates X (km)	-5.5	0.3
North coordinates Y (km)	4.3	0.5
Depth Z (km)	-10.0	0.3
Strike α (°)	129.2	1.5
Dip δ (°)	62.9	2.3
Volume change ΔV (10^6 m^3)	47.6	3.6

Supplementary Table 4. Optimal parameters of the dyke intrusion given by the time-step inversion of a set of 5 cumulative descending stacks (Supplementary Figure 2).

Parameter	20171003	20171015	20171027	20170811	20170820
East coordinates X (km)	-3.5	-4.0	-1.9	-3.3	-4.1
North coordinates Y (km)	1.8	3.3	2.5	1.9	3.0
Volume change ΔV (10^6 m^3)	35.7	49.8	58.1	63.4	42.4

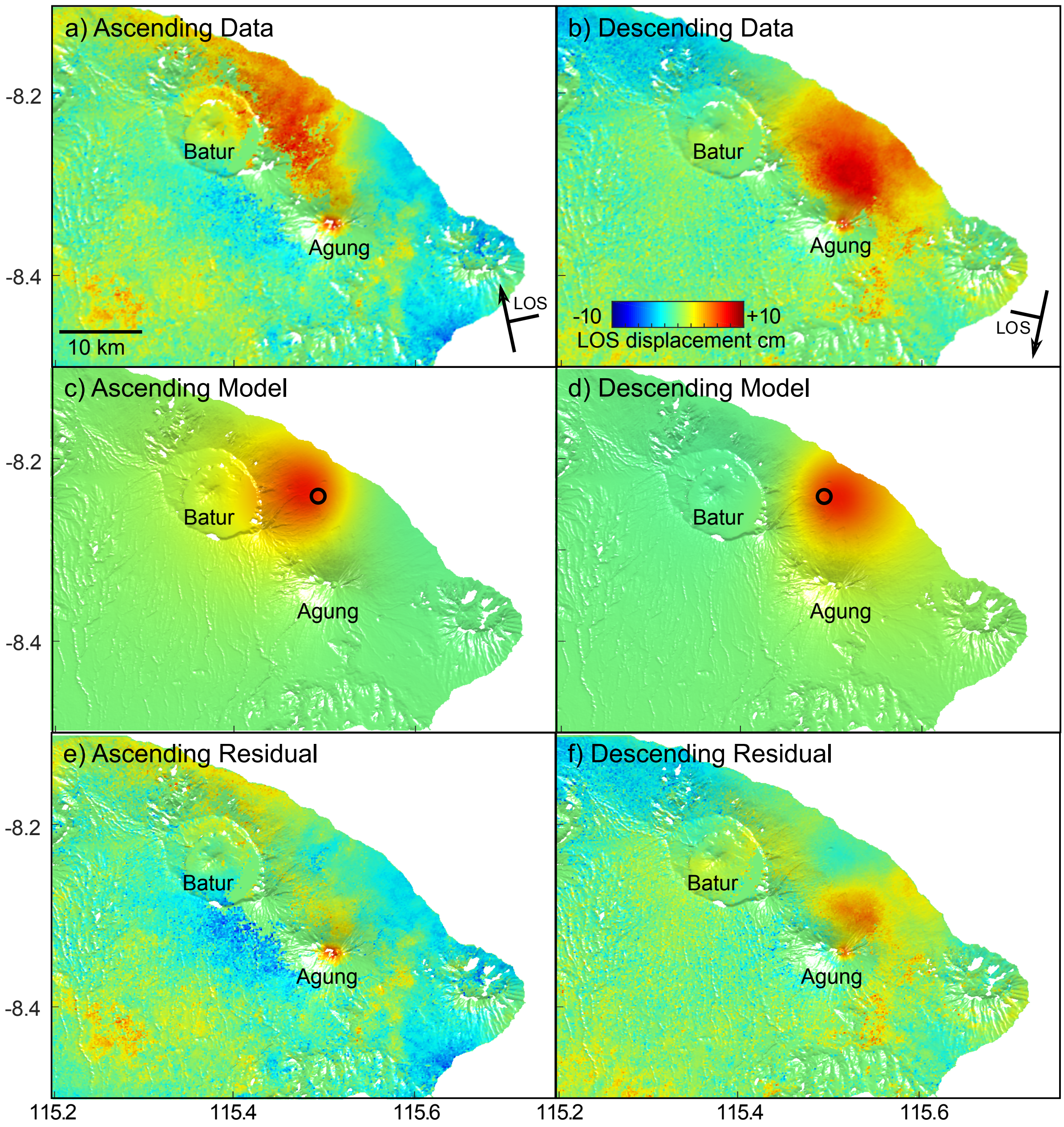

Supplementary Figure 1. a,b) Ascending and Descending pre-eruptive surface deformation. c,d) Best-fit model displacement for a spherical reservoir (black circle) e,f) Residual displacements obtained after removing the signal induced by the best-fit spherical model. Root-mean square errors are 1.6 cm and 1.2 cm for ascending and descending, respectively.

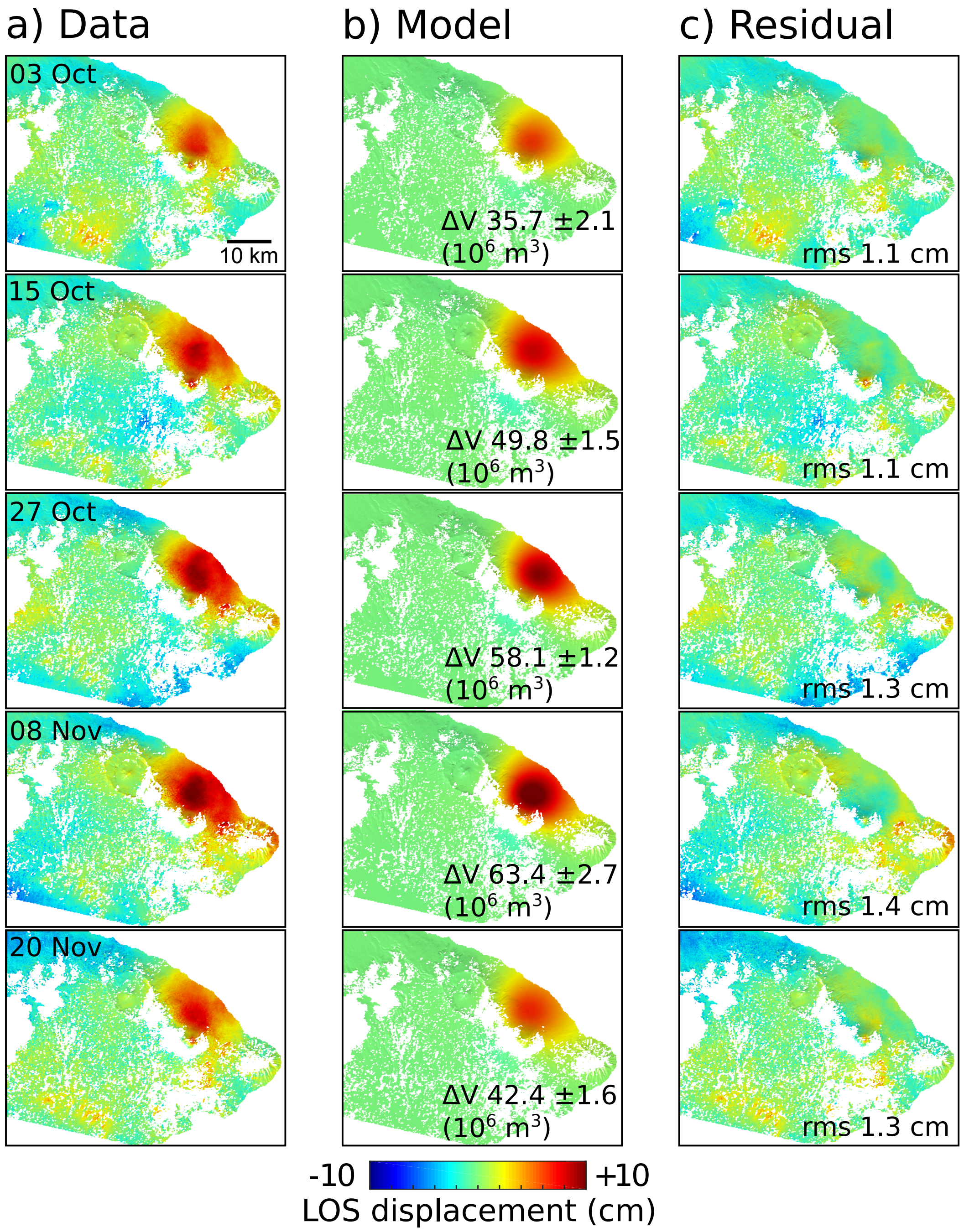

Supplementary Figure 2. Spatio-temporal evolution of ground deformation signals (descending track) at Agung from October to the onset of the eruption; left: stacked unwrapped interferogram; centre: best-fit intrusion model; right: residual calculated by the difference between the data and the corresponding best-fit model. Root mean square errors are indicated for each time-step.
